# Transcutaneous Tibial Nerve Stimulation for Quality-of-Life Improvement and Sleep Deficiency in Women with Primary Dysmenorrhea: A Randomized Clinical Trial

**DOI:** 10.3390/jcm13206262

**Published:** 2024-10-20

**Authors:** Marta Correyero-León, Javier Calvo-Rodrigo, Jorge Juan Alvarado-Omenat, Rocío Llamas-Ramos, Inés Llamas-Ramos

**Affiliations:** 1 Department of Nursing, Universidad de Valladolid, 47011 Valladolid, Spain; marta.correyero.leon@gmail.com; 2 Hospital Central de la Defensa Gómez Ulla, 28047 Madrid, Spain; calvorodrigoj@gmail.com; 3 FisioSport Salamanca, 37008 Salamanca, Spain; jjao@usal.es; 4 Department of Nursing and Physiotherapy, Universidad de Salamanca, 37007 Salamanca, Spain; inesllamas@usal.es; 5 Instituto de Investigación Biomédica de Salamanca (IBSAL), 37007 Salamanca, Spain; 6 University Hospital of Salamanca, 37007 Salamanca, Spain

**Keywords:** primary dysmenorrhea, posterior tibial nerve stimulation, quality of life, sleep quality, physiotherapy, randomized clinical trial

## Abstract

**Background**: Primary dysmenorrhea is a leading cause of chronic cyclic pelvic pain, contributing to a reduced quality of life and sleep disturbances in women. The objective of this study was to assess the effectiveness of transcutaneous tibial nerve stimulation (TTNS) in improving the quality of life, sleep, and overall health perceptions of participants compared to a control group of women with dysmenorrhea over short-term, medium-term, and long-term periods. **Methods**: A single-blind, controlled clinical trial was conducted, with participants randomly assigned to an experimental group (receiving TTNS) or a control group (receiving sham TTNS). Both groups underwent 12, weekly 30 min sessions using the NeuroTrac™ PelviTone electrostimulation device. Outcomes related to quality of life, sleep deficiency, and overall improvement were evaluated at three time points: short-term (post-treatment), medium-term (1–3 months), and long-term (6 months). **Results**: Of the 61 participants initially randomized (31 in the experimental group and 30 in the control group), 55 completed the study and were included in the final analysis. A statistically significant improvement was observed in the experimental group in both physical and mental health components, as measured by the SF-36v2^®^ questionnaire, following 12 weeks of intervention, compared to the control group, persisting 6 months after the intervention. Additionally, statistically significant differences in overall improvement were noted between the two groups, as measured by the PGIC questionnaire at the end of treatment (*p* = 0.0103) and 6 months post-treatment (*p* = 0.0432). **Conclusions**: TTNS appears to be a safe and effective strategy for enhancing quality of life and overall health in women with PD, potentially reducing the reliance on pharmacological treatments or more invasive methods.

## 1. Introduction

Primary dysmenorrhea (PD) is one of the major causes of chronic cyclic pelvic pain, leading to reduced quality of life and sleep disturbances in women [1,2,3]. According to data from the World Health Organization, up to 94% of young women aged 10 to 20 and 8.8% of women aged 19 to 41 experience menstrual pain [4,5]. Among those affected, approximately 10–25% will suffer from severe pain that incapacitates them from daily activities [3]. PD is characterized by cramping or aching pain in the lower abdomen and suprapubic region, which may radiate to the back and thighs, and typically lasts for the first 24–48 h of menstruation [2,3,6]. This pain appears to be mediated by elevated levels of prostaglandins, particularly PGF2α and PGE2, in the menstrual fluid. These prostaglandins lead to increased uterine contractions, reduced uterine blood flow, and subsequently cause hypoxia and pain [1,2,3]. It is often associated with other symptoms such as dizziness, nausea, vomiting, diarrhea, fatigue [3,7,8], headache, loss of appetite, edema, a feeling of heaviness, insomnia, negative psychological effects (anxiety, depression, irritability, nervousness, hopelessness, apathy, dysphoric feelings and lack of concentration) [2,8,9,10], and, in severe cases, syncope or fainting [9]. Dysmenorrheic pain and its associated symptoms have an immediate negative impact on quality of life, with women suffering from PD showing significantly lower quality of life, worse mood, and sleep disturbances during menstruation compared to their pain-free follicular phase and compared to the menstruation phase of women without pain [2,3]. In many cases, this situation leads to school and work absenteeism, as well as limitations in social, academic, and sports activities [7,8,10,11]. It is estimated that between 30% and 50% of women with PD miss school or work activities at least once per menstrual cycle [12].

The pharmacological treatment of PD is based on anti-inflammatory drugs (NSAIDs) or hormonal therapy [2,3]. However, other conservative treatments with minimal side effects are also being developed, such as various physiotherapy techniques, although these approaches have been little studied. Various conservative non-pharmacological treatments, including transcutaneous electrical nerve stimulation (TENS) [2,13,14], kinesiotaping [14,15], and physical exercise, have been suggested in the literature [14,16,17]. These approaches are recommended as complementary therapies for short-term reductions in menstrual pain. Promising results have been found in improving short-term quality of life [8,9,16,18,19] and sleep deficiency [19,20] with exercise-related techniques, although with low-quality evidence.

This study suggests using transcutaneous tibial nerve stimulation (TTNS), which involves stimulating the posterior tibial nerve in the superomedial area of the ankle. Stimulation of the posterior tibial nerve (L4-S3) is expected to restore the balance between inhibitory and excitatory impulses regulating the function of pelvic organs at the spinal cord level, as this nerve shares spinal levels with the hypogastric sympathetic plexus (L4-L5) and the pelvic parasympathetic plexus (S2-S4) [21,22]. This mixed nerve, which contains motor and sensory fibers, originates from a branch of the sciatic nerve and is involved in bladder innervation. Afferent nerve stimulation can activate inhibitory sympathetic neurons in the pelvic ganglia, thereby interrupting detrusor contraction. According to some authors, neuromodulation occurs through a convergence of signals that generate a long spinal reflex, activating inhibitory sympathetic neurons while suppressing the activity of excitatory parasympathetic neurons. This process helps restore the sensation of bladder fullness and reduces detrusor contraction by interfering with the afferent activity of the urethral sphincter [23,24,25].

This technique was first applied in 1983 by McGuire et al. [26] and later in 1999 by Stoller [27] for treating an overactive bladder. Since then, it has developed into two main application methods: transcutaneous and percutaneous. Positive outcomes have been reported in reducing symptoms of conditions like overactive bladder [28,29,30], fecal incontinence [31,32], and chronic pelvic pain [33,34]. The literature suggests the reduction in pain may be caused by factors such as the afferent A delta fibers and C fibers inhibitions by stimulation of somatic fibers (gate control theory), increased endorphin levels at the spinal level, or decreased c-fos expression in the central nervous system [34,35,36]. Additionally, we hypothesized that stimulation of the posterior tibial nerve could help rebalance contraction signals in the uterine myometrium, like the rebalancing of micturition impulses in the bladder, thereby reducing uterine hypercontractility and alleviating menstrual pain. This reduction in pain could improve the quality of life and sleep for women with PD. However, in the literature there are no specific studies which investigate the treatment of PD with TTNS. Therefore, the objective of this study was to determine whether TTNS can improve quality of life, sleep, and overall improvement, compared to a control group of women with dysmenorrhea, over short-term, medium-term, and long-term periods, without causing harmful effects.

## 2. Materials and Methods

### 2.1. Study Design

A randomized, single-blind, controlled clinical trial was developed to assess the efficacy and safety of TTNS in improving quality of life and sleep deficiency in women with PD. This research was carried out at a private physiotherapy center in Valladolid, Spain. The study received approval from the Ethics Committee for Drug Research of the Valladolid East Health Area, Hospital Clínico Universitario de Valladolid, located at Hospital Clínico Universitario de Valladolid, Av. Ramón y Cajal, 3, 47003 Valladolid (Spain), on 12 December 2019, under the approval code CASVE-NM-19-423. The study has followed the Helsinki and CONSORT guidelines, with all participants providing informed consent. The trial is registered on ClinicalTrials.gov with the ID: NCT04896814; https://clinicaltrials.gov/study/NCT04896814 (accessed on 30 August 2024).

### 2.2. Sample

Participants who met the following criteria were eligible for inclusion in the study: women aged 18 to 43 with regular menstrual cycles [9,15,17,20] who experienced pain rated between 4 and 10 on the Visual Analog Scale (VAS) [9,15] in the suprapubic region, abdomen, lower back, perineum, and/or inner thighs during half of their yearly menstrual cycles (as a minimum) and/or in the last three cycles. Exclusion criteria included women with an implanted intrauterine device or those undergoing hormonal treatment [9,15,20,37]; individuals diagnosed with secondary dysmenorrhea by their gynecologist within the last 18 months (such as endometriosis or ovarian cysts) [9,12,15,20]; women who have had surgeries in abdominal or pelvic regions during the study or who have given birth in the previous 6 months [12,15]; having skin lesions, including scars or erosions, on the inner upper aspect of the ankles; pregnant women [12,15,18,20]; and those with pacemakers, uncorrected coagulopathies, severe comorbid conditions, cancer (currently or in the past 5 years), severe mental disorders, or neuropathies in lower limbs [17,18,38,39]. Additionally, women who have received physiotherapy treatment for this condition within one month prior to the study will also be excluded [39]. Throughout the study, patients were allowed to take NSAIDs at their usual doses and frequencies. The patients recorded each NSAID in a diary, including the type and amount taken during each day of their menstruation, as well as the degree of pain relief experienced with each dose.

G-Power program was used to determine the sample size and based on data from previous studies [12,37,38,40,41]. An alpha level of 0.05 and a beta risk of 0.2 were considered for a bilateral contrast. To detect a difference of 1.3 or more in peak VAS, 27 participants were needed in each group. The calculation assumed a common standard deviation of 1.6 and accounted for an estimated 10% dropout rate.

### 2.3. Randomization

All participants were allocated to either the experimental group or the control group randomly (1:1 ratio). The Random Number Generator Pro software (version 1.76) was used for the randomization process. The group assignments were concealed from the researcher, who was unaware of which group each participant would be placed in at the time of their inclusion in the study. The participants themselves also did not know which group they had been assigned to.

### 2.4. Procedure

Patient recruitment and randomization took place between 25 May and 31 May 2021. The baseline assessments were carried out during June and July 2021, with the interventions conducted from 1 August to 31 October 2021. Short- and medium-term follow-up occurred between 1 November 2021 and 30 April 2022.

The study consisted of 4 phases: an initial interview, where the women decided to participate in the study, signed an informed consent form, and completed a medical history sheet; an initial evaluation phase lasting 2 months, during which the participants’ baseline statuses were assessed; an intervention phase lasting 3 months, during which the intervention was carried out; and a reevaluation phase lasting 6 months. The intervention time in the study was based on similar nerve stimulation studies [23,34,35,42,43,44].

In the first meeting (30 min session), the characteristics of the study were explained to the participants. Participants signed an informed consent document, and they completed a medical history form for participation. This clinical history included: demographic characteristics, lifestyle habits, general medical history, gynecological, obstetric and menstrual history, and finally the menstrual pain history.

During the assessment phase, many questionnaires assessing the variables of “quality of life” and “sleep quality” were administered over two consecutive menstrual periods. The questionnaires were provided to the women during this first interview, and they were self-administering them throughout the following two menstrual cycles. The goal of this stage was to establish the initial status of the participants concerning the studied variables.

In the intervention phase, the participants received treatment and continued to complete the same questionnaires during this phase. Additionally, upon completion of the intervention, participants were asked to fill out a questionnaire to evaluate their “overall improvement.” After each treatment session, the participants completed a side effects questionnaire.

In the reassessment phase, the identical questionnaires were filled out one month after treatment, as well as at three and six months following the end of treatment. Furthermore, at the conclusion of the six month follow-up period, patients were asked to complete the harmful effects questionnaire and the satisfaction and overall improvement scales one more time.

The development of the study, the procedures description, and the variables assessed are explained in the manuscript published by Correyero-León et al., 2023 [44].

**Figure 1:**
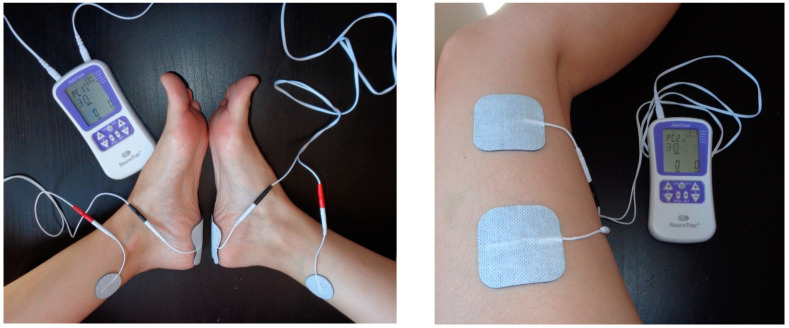
Intervention for experimental and control group.

The study’s intervention was divided into two groups. The experimental group underwent TTNS treatment, while the control group experienced a fake current applied outside the posterior tibial nerve area. The two groups attended sessions of 30 min once a week for a period of 12 weeks. The NeuroTrac™ PelviTone electrostimulation device (Verity Medical, Romsey—Hampshire, UK) was used for the treatments (Figure 1).

### 2.5. Data Collection and Outcomes

The data were gathered through self-administered questionnaires and then randomized using SPSS software version 26 (IBM Corporation, Armonk, NY, USA).

Several variables were recorded during the study. The SF-36v2^®^ Health Questionnaire was chosen to assess the physical and mental aspects related to quality of life [45,46]. A higher score on the SF-36v2^®^ indicates better health. The Pittsburgh Sleep Quality Index (PSQI^®^) (University of Pittsburgh, EEUU) was be used to evaluate sleep deficiency [47,48]. A higher score on the PSQI^®^ reflects poorer sleep quality. The patients should have completed both questionnaires on the final day of their menstrual period throughout the entire study. These data were collected during an initial two month phase to assess the initial status of the women, once a month during the treatment (short term), and then at four weeks, three months (medium term), and six months (long term) after the intervention concluded.

On the other hand, the overall improvement was evaluated using the Patient’s Global Impression of Change Questionnaire (PGIC). This questionnaire is based on 7 items from which the patient must choose the option that comes closest to the improvement obtained with this intervention. The higher the score, the worse the impression of change [49]. The patient must complete it when the treatment finishes and at the end of the reevaluation. In addition, using a Likert scale, patients were asked to provide their subjective assessment of the intervention’s success and whether they would like to continue treatment to maintain the achieved goals in a hypothetical situation. The scale qualitatively expresses the degree of agreement or disagreement with the intervention.

Potential adverse reactions to the treatment were recorded, and patients were asked to complete a brief questionnaire created by the researcher after each treatment session, with a follow-up 11 months after the start, so that potential side effects can be evaluated both immediately after the intervention (short term) and at the end of the reassessment (long term), with the aim of determining if any effects appeared over time. TTNS is considered a type of simple peripheral stimulation that is minimally invasive, easy to apply, and well tolerated by patients, with no notable side effects reported [23,25,34,35,36]. The shape of the electrical impulse affects the comfort perceived by the patient, with the symmetrical shape being referenced as the most comfortable for patients [50,51]. The most common adverse effect of electrotherapy is burns, which are virtually exclusive to galvanic current, which was not used in this study [51,52]. However, general precautions to avoid burns during electrotherapy were always ensured [52]: apply only to intact skin without lesions, excoriations, scars, pimples, recent hair removal, etc.; maintain the electrodes in good condition; ensure proper electrode fixation; and use the correct dosage.

### 2.6. Statistical Analyses

SPSS software version 26 (IBM Corporation, Armonk, NY, USA) was used to analyze data. Quantitative variables were presented as mean and standard deviation, while qualitative variables were expressed as counts and percentages. The Shapiro–Wilk test was used to assess normality. A Student’s *t*-test was chosen to establish comparisons between the two groups. To evaluate the intervention effects between groups, a repeated measures two-factor analysis of variance (rANOVA) was employed, examining differences in outcomes over time. This considered initial and post-intervention as within-subject factors and group (experimental and control) as between-subject factors. A *p*-value of less than 0.05 was set as the threshold for statistical significance.

## 3. Results

**Figure 2:**
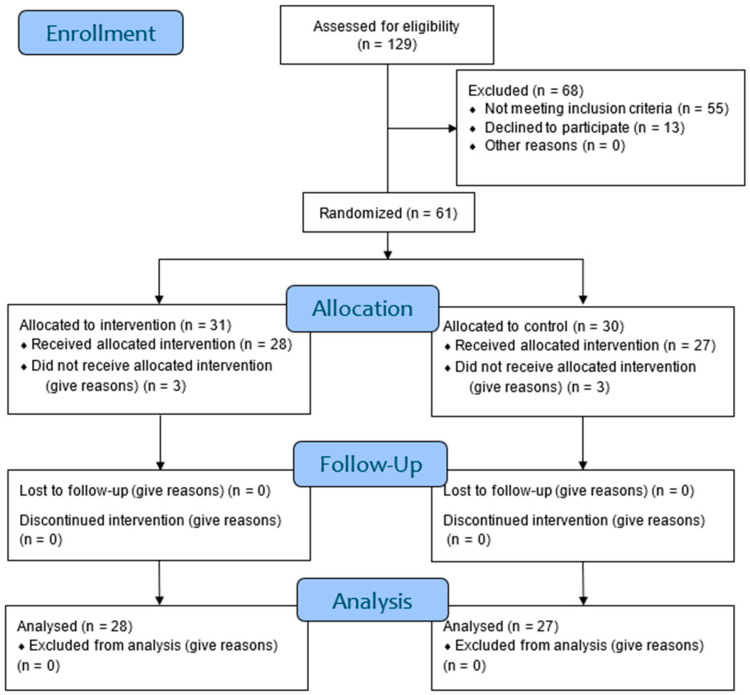
Consolidating standards of reporting trials (CONSORT) flow diagram [44].

Out of the 129 participants initially recruited, 61 were randomized, with 31 allocated to the experimental group and 30 women to the control group. Among these, 4 participants withdrew during the assessment phase without completing it, and 2 finalized phase 2 but discontinued during the intervention phase. There were no dropouts during the reassessment phase. Finally, 55 participants finished the entire clinical trial and were included in the data analysis. No dropouts occurred during the phase 4 follow-up. Figure 2 illustrates the participants’ flow diagram recruitment and follow-up.

Table 1 provides a summary of the baseline characteristics of the study participants. The participants ranged in age from 18 to 44 years. The average age was 28.4 years (SD 7.1) in the control group and 25.3 years (SD 5.6) in the experimental group. Both groups were comparable in terms of baseline demographic characteristics and initial status.

A statistically significant improvement was found in the experimental group in the physical (*p* = 0.008) and mental components (*p* > 0.047) measured with the SF-36v2^®^ in the within-group analysis after 12 weeks of intervention compared to the control group. However, the rANOVA analysis (normal sample distribution) did not show statistically significant group-by-time interactions for the physical (*p* = 0.08) and mental (*p* = 0.18) components measured with the SF-36v2^®^.

After the end of the 12-week intervention, the rANOVA analysis did not show statistically significant differences in sleep improvement, measured with the PSQI^®^ (*p* = 0.25) (Table 2).

In the medium-term to long-term (reassessments at 1, 3, and 6 months), the rANOVA analysis did not show statistically significant results in the improvement of physical (*p* = 0.57) and mental (*p* = 0.60) components according to the SF-36v2^®^ or in sleep improvement according to the PSQI^®^ (*p* = 0.16). However, significant improvements were maintained in the within-group analysis for both SF-36v2^®^ (physical components *p* = 0.01; mental components *p* < 0.001) related to quality of life in the experimental group after 6 months of re-evaluation compared to the control group (Table 3).

On the other hand, statistically significant improvements were found between the two groups in overall improvement as measured by the PGIC questionnaire at the end of treatment (*p* = 0.01) and at 6 months post-treatment (*p* = 0.04) (Table 4). Immediately after the intervention, 40% of the women in the experimental group, compared to 23.33% of the women in the control group, agreed to continue with the treatment. After the 6 month reassessment, 30% of the women in the experimental group, compared to 13.33% of the women in the control group, agreed to continue with the treatment.

No harmful effects related to the intervention with TTNS were reported in the short term or over the medium to long term.

## 4. Discussion

The objective of this study was to test the TTNS effects on improving quality of life and alleviating sleep deficiency, as well as to assess the global impression of change in patients with PD.

To the best of our knowledge, this is the first randomized clinical trial which explored these effects, compared them to a control group, and demonstrated statistically significant results in both short- and long-term quality of life improvement, as well as a statistically significant enhancement in the overall condition as perceived by the participants.

Women with PD frequently report pain and other symptoms associated with menstruation, leading a high percentage of patients to experience limitations in daily activities, particularly in sports and leisure activities, followed by academic and occupational activities. It has been observed that all these factors negatively impact the quality of life and sleep quality in the short, medium, and long term, affecting both the physical and emotional domains of these patients. Therefore, these variables were included in the present study.

Different publications in the literature have examined the quality of life of patients with PD in relation to various non-invasive physiotherapeutic techniques [9,12,15,39,53,54,55,56]. In the present study, statistically significant differences were found in the experimental intragroup comparison for the variables measuring quality of life at 8 and 12 weeks of treatment, improvements that are also observed in studies evaluating other electrotherapeutic techniques, such as that of Dögan et al. [15]. Conversely, other studies did not state statistically significant improvements in the experimental group regarding quality of life [39,53]. It is interesting to compare these variables with the control group, as no statistically significant improvements were observed in this group at any short, medium, or long-term intervals in intragroup comparisons. This finding is particularly relevant because, despite not finding statistically significant improvements in the between-group analysis, this change did occur in the experimental group. These improvements persisted over time and continued to be evident six months post-treatment in the present study, which was not observed in long-term analyses in the other studies evaluated.

Only two articles have evaluated sleep quality in women with PD [8,20]. This aspect was included in the present study since sleep quality may be impaired by pain (e.g., nighttime awakenings). Moreover, elevated progesterone levels physiologically produce more frequent awakenings during the night, which would negatively impact sleep quality [57,58]. In the publication by Kirmizigil et al. [20], a statistically significant improvement in the total PSQI^®^ score was observed in the experimental intragroup comparison, although these differences were not found in the present research.

Furthermore, statistically significant differences were found in global improvement and treatment satisfaction measured by the PGIC questionnaire at the end of the intervention, and six months post-intervention, similar to the findings in the studies by Dögan et al. [15] and Gaubeca-Gilarranz et al. [12]. These statistically significant differences in between-group comparisons confirm the trend toward improvement in the experimental group, observed in the intragroup analysis of quality of life, which is subjectively corroborated by the participants’ confirmation of their perceived improvement compared to the control group participants. Therefore, it could be hypothesized that neuromodulation of uterine contractions reduced pain in the participants, leading to an improved quality of life and better self-perception of their health status.

The manuscript has limitations; there was no blinding of the investigator implementing the TTNS intervention, the intake of NSAIDs during the treatment was concurrent, and pain relief rather than pain origin was addressed. This is further complicated by the absence of tools to measure prostaglandin levels or uterine contractility.

In addition to the quality of life and sleep deficiencies variables, the treatment primarily aims to alleviate or reduce pain rather than eliminate it by treating its underlying cause [59]. The observed pain reduction may be linked to the stimulation of the posterior tibial nerve, which potentially affects the pelvic nerves and modulates nerve transmission, thus reducing uterine hypercontractility. This also aligns with pain relief mechanisms such as the gate control theory, among others. However, in future studies, treatments focusing on the pain origin must be considered.

## 5. Conclusions

The findings of the present study indicate that TTNS may be an effective and safe approach to improving the quality of life and overall health in women with PD, potentially reducing the need for pharmacological treatments or more invasive methods. However, further research with a longer sample size is necessary to confirm these benefits over the medium to long term, along with studies to compare and determine which techniques are most effective.

## Figures and Tables

**Table 1:** Study population baseline characteristics.

	Control Group	Experimental Group
Age (years)	28.4 (7.1)	25.3 (5.6)
BMI (kg/cm^2^)	22.14 (3.22)	22.39 (2.85)
Children n (%)		
0	23 (85.2)	26 (92.9)
1	2 (7.4)	1 (3.6)
2	1 (3.7)	1 (3.6)
3	1 (3.7)	0 (0.0)
Tobacco n (%)		
No	23 (85.2)	22 (78.6)
Yes	2 (7.4)	2 (7.1)
Occasionally	1 (3.7)	2 (7.1)
Former smoker	1 (3.7)	2 (7.1)
Alcohol n (%)		
No	2 (7.4)	3 (10.7)
Yes	2 (7.4)	1 (3.6)
Occasionally	23 (85.2)	24 (85.7)
Physical Activity n (%)		
No	3 (11.1%)	5 (17.9)
Yes	16 (59.6)	17 (60.7)
Occasionally	8 (29.6)	6 (21.4)
Cycle Duration n (%)		
21–27 days	6 (22.2)	4 (14.3)
28–31 days	16 (59.3)	21 (75.0)
32–35 days	5 (18.5)	3 (10.7)
Menstruation Duration n (%)		
1–3 days	1 (3.7)	6 (21.4)
4–6 days	23 (85.2)	19 (67.9)
7–9 days	3 (11.1)	3 (10.7)
Fatigue n (%)		
Never	4 (14.8)	7 (25.0)
Occasionally	12 (44.4)	12 (42.9)
Very often	5 (18.5)	6 (21.4)
Always	6 (22.2)	3 (10.7)
Insomnia n (%)		
Never	8 (29.6)	10 (35.7)
Occasionally	11 (40.7)	10 (35.7)
Very often	5 (18.5)	5 (17.9)
Always	3 (11.1)	3 (10.7)
Depression n (%)		
Never	19 (70.4)	19 (67.9)
Occasionally	7 (25.9)	7 (25.0)
Very often	1 (3.7)	0 (0.0)
Always	0 (0.0)	2 (7.1)
Anxiety n (%)		
Never	15 (55.6)	18 (64.3)
Occasionally	10 (37.0)	7 (25.0)
Very often	0 (0.0)	2 (7.1)
Always	2 (7.4)	1 (3.6)
Irritability n (%)		
Never	5 (18.5)	2 (7.1)
Occasionally	10 (37.0)	13 (46.4)
Very often	7 (25.9)	8 (28.6)
Always	5 (18.5)	5 (17.9)

**Table 2:** Mean values obtained in the pre-treatment and post-treatment evaluations of the aspects examined by the SF-36v2^®^ and PSQI^®^, in the short term.

	Pre-Assessment		8 Weeks		Intragroup Analysis 1		12 Weeks (3 Months)		Intragroup Analysis 2		Analysis Between Groups rANOVA	
Variable	Mean (±SD)		Mean (±SD)		*p* Value		Mean (±SD)		*p* Value		F	*p* Value
	EG (n = 28)	CG (n = 27)	EG (n = 28)	CG (n = 27)	EG (n = 28)	CG (n = 27)	EG (n = 28)	CG (n = 27)	EG (n = 28)	CG (n = 27)	EG (n = 28) vs. CG (n = 27)	
physical components (SF-36v2^®^)	49.23 (5.45)	51.86 (6.04)	52.29 (5.81)	53.91 (3.89)	0.001 *	0.078	51.51 (5.06)	51.77 (5.23)	0.008 *	<0.001 *	3.11	0.084
mental components (SF-36v2^®^)	39.56 (10.16)	40.43 (10.98)	45.71 (8.40)	42.26 (10.07)	<0.001 *	<0.001 *	46.97 (8.34)	43.68 (7.89)	0.047 *	0.124	1.82	0.183
sleep quality (PSQI^®^ total)	5.25 (2.84)	6.26 (3.47)	3.93 (2.91)	5.78 (3.0)	<0.001 *	<0.001 *	3.93 (2.51)	5.67 (2.96)	<0.001 *	<0.001 *	1.38	0.246

SD: standard deviation, EG: experimental group, CG: control group; Intragroup analysis 1: analysis between the data before the intervention and the eight weeks of intervention; Intragroup analysis 2: analysis between the data before the intervention and the twelve weeks of intervention; *: Statistically significant differences; rANOVA: repeated measures two-factor analysis of variance; SF-36v2^®^: SF-36v2^®^ Health Questionnaire; PSQI^®^: Pittsburgh Sleep Quality Index.

**Table 3:** Mean values obtained in the pre-treatment and post-treatment evaluations of the aspects examined by the SF-36v2^®^ and PSQI^®^, in the medium-long term.

	Pre-Assessment		4 Months		Intragroup Analysis 1		6 Months		Intragroup Analysis 2		9 Months		Intragroup Analysis 2		Analysis Between Groups rANOVA	
Variable	Mean (±SD)		Mean (±SD)		*p* Value		Mean (±SD)		*p* Value		Mean (±SD)		*p* Value		F	*p* Value
	EG (n = 28)	CG (n = 27)	EG (n = 28)	CG (n = 27)	EG (n = 27)	CG (n = 27)	EG (n = 28)	CG (n = 27)	EG (n = 28)	CG (n = 27)	EG (n = 28)	CG (n = 27)	EG (n = 28)	CG (n = 27)	EG (n = 28) vs. CG (n = 27)	
physical components (SF-36v2^®^)	49.23 (5.45)	51.86 (6.04)	52.48 (4.37)	53.17 (5.19)	0.13 *	<0.001 *	52.51 (4)	52.04 (5.08)	0.001 *	0.023 *	50.97 (5)	52.92 (5.65)	0.013 *	0.114	0.33	0.567
mental components (SF-36v2^®^)	39.56 (10.16)	40.43 (10.98)	45.67 (6.94)	42.11 (8.30)	0.011*	0.024 *	44.89 (7.40)	42.21 (9.64)	<0.001 *	0.019 *	44.80 (9.61)	43.89 (9.33)	<0.001 *	0.085	0.28	0.601
sleep quality (PSQI^®^ total)	5.25 (2.83)	6.26 (3.47)	4.21 (2.81)	5.04 (3.44)	<0.001 *	<0.001 *	3.79 (2.35)	5 (3.10)	<0.001 *	<0.001 *	3.75 (2.38)	5.48 (3.03)	<0.001 *	<0.001 *	2.04	0.159

SD: standard deviation, EG: experimental group, CG: control group; Intragroup analysis 1: analysis between the data before the intervention and the eight weeks of intervention; Intragroup analysis 2: analysis between the data before the intervention and the twelve weeks of intervention; Intragroup analysis 3: analysis between the data before the intervention and the reevaluation at the six months after completing the intervention; *: Statistically significant differences; rANOVA: repeated measures two-factor analysis of variance; SF-36v2^®^: SF-36v2^®^ Health Questionnaire; PSQI^®^: Pittsburgh Sleep Quality Index.

**Table 4:** Mean values obtained in the post-treatment evaluations of the aspects examined by the PGIC questionnaire, in the medium-long term.

	Experimental Group	Control Group	*p* Value
PGIC post-treatment (3 months)	2.83 (0.99)	3.47 (0.63)	0.010
PGIC post-evaluation (9 months)	3.03 (0.93)	3.57 (0.90)	0.043

## Data Availability

The data presented in this study are available on request from the corresponding author.
